# Detection of Prosthetic Knee Movement Phases via In-Socket Sensors: A Feasibility Study

**DOI:** 10.1155/2015/923286

**Published:** 2015-04-05

**Authors:** Amr M. El-Sayed, Nur Azah Hamzaid, Kenneth Y. S. Tan, Noor Azuan Abu Osman

**Affiliations:** ^1^Department of Biomedical Engineering, Faculty of Engineering, University of Malaya, 50603 Kuala Lumpur, Malaysia; ^2^Mechatronics Section, Mechanical Engineering Department, Faculty of Engineering, Assiut University, Assiut 71516, Egypt

## Abstract

This paper presents an approach of identifying prosthetic knee movements through pattern recognition of mechanical responses at the internal socket's wall. A quadrilateral double socket was custom made and instrumented with two force sensing resistors (FSR) attached to specific anterior and posterior sites of the socket's wall. A second setup was established by attaching three piezoelectric sensors at the anterior distal, anterior proximal, and posterior sites. Gait cycle and locomotion movements such as stair ascent and sit to stand were adopted to characterize the validity of the technique. FSR and piezoelectric outputs were measured with reference to the knee angle during each phase. Piezoelectric sensors could identify the movement of midswing and terminal swing, pre-full standing, pull-up at gait, sit to stand, and stair ascent. In contrast, FSR could estimate the gait cycle stance and swing phases and identify the pre-full standing at sit to stand. FSR showed less variation during sit to stand and stair ascent to sensitively represent the different movement states. The study highlighted the capacity of using in-socket sensors for knee movement identification. In addition, it validated the efficacy of the system and warrants further investigation with more amputee subjects and different sockets types.

## 1. Introduction

An amputee user's locomotion phase detection in the field of transfemoral prosthesis system is still undergoing extensive research, especially detection originating directly from the users themselves. In general, a transfemoral prosthesis system has always been mechanically based in which the user had to adapt his gait pattern to accommodate the passive behavior of the prosthesis. Having knee joint across the prosthesis increased the complexity of the system but over the years, advancement of passive adaptive and active prosthetic knee has resulted in improved systems and designs for transfemoral amputees [1–3].

Nowadays, active prosthetic knee systems utilized sensors at certain locations around the prosthetic knee to measure specific parameters. Most of the current sensory systems in the development of prosthetic knee devices are usually located away from the knee axis and the muscles themselves. Such sensors measure parameters such as force, torque, position, velocity, and phase transitions for appropriate control decisions. The information derived from these mechanical sensors was used to derive the instantaneous state of movement to further control the prosthesis system. However, more accurate information about the user's instantaneous state of movement could be derived from the sensors if they are located closest to the user peripherals or nearby the knee joint axis itself. Optimal location of the sensors in a prosthetic knee system may provide better deduction capability of the prosthesis to improve user interaction and performance during daily activities, as the accuracy gained from better sensor placement could reduce the complexity of the knee control.

The identification of the different parameters during prosthetic knee movement is essential to control the knee. For example, the most critical input to be addressed during a transfemoral prosthesis controlled gait is the foot position, either on or off the ground, and this was determined from the angle, torque, and force sensors measurements. As the transition between gait phases is crucial for the control of active knee, such inertial sensors are used to recognize the transitions between the gait phases [4–6]. A magnetorheologic fluid actuated prosthetic knee used a strain gage sensors as an axial force sensors [1], sensor that is placed nearby the knee axis to detect force and torque [1]. The axial force sensors measured the force applied to the prosthetic knee from the ground in the longitudinal direction of the knee. Measurement of the knee torque was conducted by classifying the difference between the signals of the forward and hind strain gages [1]. Other sensory mechanisms used by other developed systems were summarized and presented in Table 1. In general, all sensors were embedded into the prosthetic system to deduce the user's current and intended knee movement without measuring them directly from the socket.

Another approach that is used to gain direct input from the muscle to control the active prosthetic knee is by using the electromyography (EMG) system. Direct user interaction was enabled in an active prosthetic knee system by embedding EMG system. In systems that incorporate EMG, the sensors are positioned to detect the user's flexor and extensor muscles activities from generally the rectus femoris, vastus lateralis, vastus medialis, biceps femoris, and semitendinosus. The EMG signal was utilized to formulate the control algorithm that assists the user to control the torque during activities such as stair ascent. However, the muscle activity may be varied depending on the individual amputee's residual limb muscles or according to the amputation type and level. This may require additional adjustment to the EMG electrodes and the control system [7, 8]. Inertial sensors such as accelerometer and EMG were used in combination to identify the start of the gait by using a technique called “per leading limb condition” of the prosthetic leg during walking [9]. However, skin conditions of the transfemoral amputees may affect the use of EMG [10]. In addition, the placement of EMG onto the skin surface and inside the socket may cause skin irritation and affect the user's comfort [11]. Therefore, another approach is needed to improve the control of the active knee device by choosing proper locations of the sensory system. The suitable location of the sensors could minimize the complexity of the control scheme of the lower prosthesis.

The signals from the inertial sensors are not the only ones that may be acquired to help improve the control of the active knee prosthesis. Further investigation on other alternative signals for characterizing the prosthetic knee movement for better control of the knee prosthesis should be conducted and integrated into future system developments [12]. Alternative options that could better characterize the knee movement will aid the designer to identify multiple solutions to improve the area of active prosthetic knee development [13].

Nowadays, researchers try to involve the amputee subjects with the sensory system more closely to assist the controller decision making. Attempts are ongoing to assist the amputee subjects to interact more naturally with the sensory system by making use of the specific high pressure locations inside the socket. Other sensors placed inside the socket such as the F-socket sensor have been used in investigating the pressure around the residual limb, but they were not meant for daily integration into the socket for identifying knee movements in active transfemoral prosthesis [14]. Various kinds of pressure sensors are used to measure the pressure for both transtibial and transfemoral amputees [14, 15]. Current pressure socket measurement systems such as F-socket (Tekscan, Inc., South Boston, USA) or pressure measuring system (Novel, Germany) were used to cover the circumference of the residual limb. However, they have to include all the posterior, anterior, lateral, and medial compartments of the residual limb. Nevertheless, by selecting specific locations inside the socket, limited number of sensors could be placed to provide sufficient measurements that would help to better improve the control scheme of the active knee.

In general, we proposed that direct user signals could be collected from sensors embedded in the socket and residual limb. This study aims to embed the sensory system inside the patient's socket, as this approach will provide less additional components and practically less setup time, thus more flexibility to the patient wearing the socket. This paper presented the efficacy of embedding mechanical sensors inside the socket's internal wall for movement identification. FSR and piezoelectric sensors were placed inside the socket to achieve the aim of this study. In the proposed study, the obtained in-socket data from the interaction between the sensors and the amputee, as well as the biomechanical position of the ground reaction force acting against the sensors inside the socket due to the amputee's specific body posture, will enable the recognition of the user's leg movement as well as events of the movement. These were done by considering the signals from the sensors at different prosthetic knee movements performed by the amputee subject.

## 2. Materials and Methods 

### 2.1. Sensor Characteristics and Utilization

**Figure 1:**
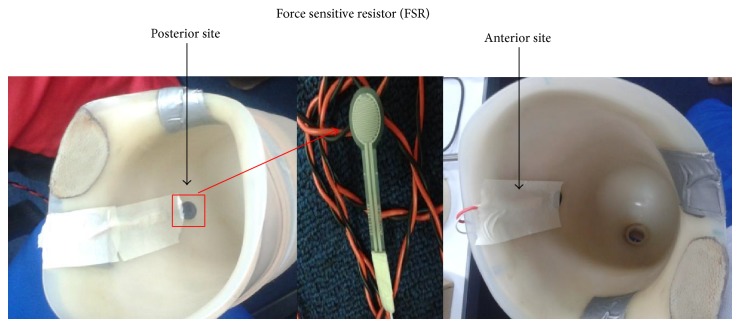
FSRs locations inside the socket during the experiment for both anterior and posterior sites.

**Figure 2:**
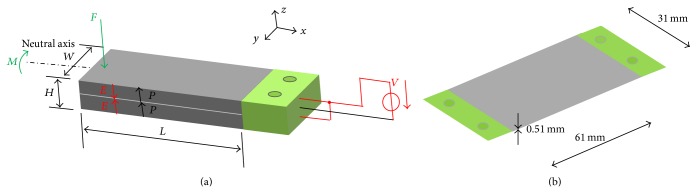
(a) Basic dimensions, extensive parameters, polarization, and applied electric field acting on the bimorph generator [16]. (b) Dimensions of the used bimorph with two fixed ends.

The adopted sensors (FSR and piezoelectric) in the current study were placed inside the socket wall (Figure 1). FSR was chosen based on its small size (1.25 mm thickness and 12.7 mm diameter) that will not affect the user comfort. Similarly the piezoelectric sensor has a configuration (Figure 2) as well as dynamic characteristics that make it suitable for such applications [16]. The sensors were tethered to transmit the data directly to the PC via wires. The minimal thickness did not affect the user's natural movements. These sensors were able to accurately characterize the knee movements during walking, stair climbing, and sit to stand.

#### 2.1.1. FSR Sensor and Piezoelectric Sensors

Two FSR sensors (Interlink Electronics 402, Interlink Electronics, USA) of sensing area diameter 12.7 mm were used in the current study based on the site that generated maximum stresses [17]. A signal conditioning circuit was built to acquire the output voltage from the FSR at a range of 0 to 3.5 volts. The output voltage from the FSR circuit was connected to a Simulink environment by using the Real-Time Windows Target Toolbox. Afterwards, a data acquisition system (Advantech PCI-1710HG, Advantech, USA) was utilized to analyze the output data from the FSR sensor.

The FSRs were placed at specific locations in the socket to effectively capture the maximum stress of the socket's area [15]. Given the small area covered by the sensor, the anatomical muscle bulge during maximum contraction was identified to determine the sensor placement in the socket. Furthermore, to ensure that the sensor is in contact with the greatest pressure point against the socket wall when the muscle contracts, investigators palpated the muscles during maximum voluntary contraction of the amputee's residual limb. This ensured that the FSR was located at a position that allowed detection of highest variation of the signal originating from high pressure at the rectus femoris and biceps femoris muscles contraction [15, 18]. Given the minimal thickness of the FSR (<1.25 mm), the FSR was secured using adhesive sticker inside the socket's wall. This eliminated the user sensational awareness about the FSR in the socket which otherwise would have affected the user's natural movements. Trials were conducted to estimate the pattern variation of three major movements, namely, (i) full stance of gait, comprising heel strike, flat foot, and toe off; (ii) stair ascent; and (iii) sit to stand. The socket with the attached in-socket FSR is presented in Figure 1.

**Figure 3:**
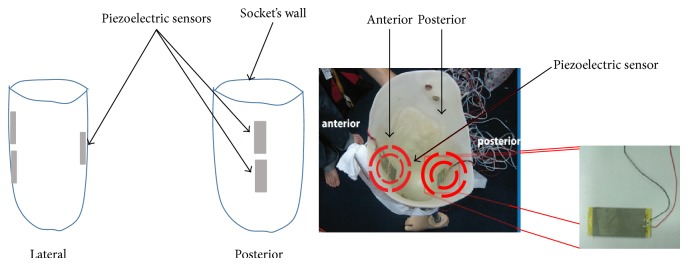
Placement of the piezoelectric sensors at both anterior and posterior sites.

Piezoelectric sensors are used to identify the knee movement and facilitate the interaction between the user and the lower prosthesis through the socket. Piezoelectric sensors have been used to provide another technique that may help in the characterization of the knee movement. In addition, it may be compared to the FSR sensors to illustrate the extent of which both of them may be practically useful for the lower limb's designer. Moreover, the captured signals from the sensor assist in the development of prosthetic knee, in terms of the control strategy during different schemes. The piezoelectric sensors in this study were also placed inside the socket wall with specially made cavity to securely attach the sensor while allowing the required piezoelectric sensor deflection (Figure 3). Basically, one of the advantages of using piezoelectric bimorph is that it does not require external power supply to operate as it is considered an active sensor. Moreover, it also can be used to harvest energy when mechanical stress is applied on the bimorph surfaces [19, 20]. Basically, it consists of two layers sandwiched by metal layer for more flexibility as shown in a bimorph configuration as in Figures 2(a) and 2(b). Bimorph sensor is one of the most widely used bender actuators in both academic studies and industrial applications [16]. When applying pressure to the surface an electrical charge appears. The amount of charge is transferred into measurable output voltage which is proportional to the amount of pressure. The piezoelectric bimorph has a good dynamic characteristics in terms of handling transient inputs; also it has a wide range of output voltage up to ±90 V as well as a bandwidth about 100 Hz [16]. In addition to, the bimorph layer has a bleed resistor that protects it from high transient voltages and mechanical shocks.

Three piezoelectric sensors were attached at specific positions [15] at the anterior distal, anterior proximal, and posterior sites of the socket in order to sense the knee movement at different phases. A third piezoelectric sensor was placed at the anterior site nearby the knee joint to collect better measurement about the joint movement [1]. Figure 3 shows the placement of the piezoelectric sensors at both anterior and posterior sites.

### 2.2. Subject Characteristics and Experiments

**Figure 4:**
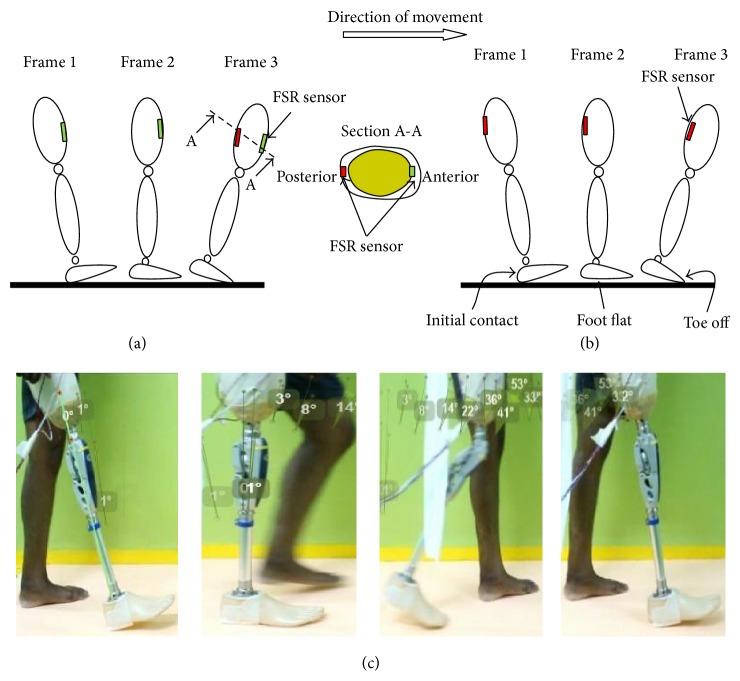
(a) Anterior FSR placement during full stance phase; (b) posterior FSR placement during full stance phase; and (c) the individual performing full stance phase (heel strike, flat foot, and toe off) while wearing the FSR instrumented socket.

**Figure 5:**
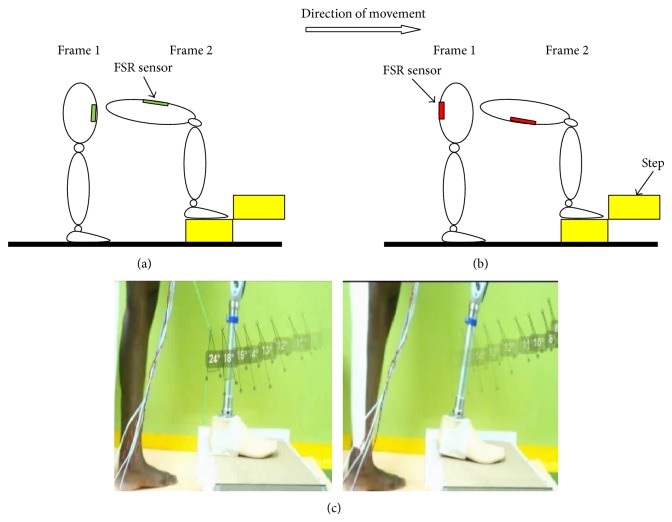
(a) Anterior FSR placement during stair ascent; (b) posterior FSR placement during stair ascent; and (c) the individual performing stair ascent while wearing the FSR instrumented socket.

**Figure 6:**
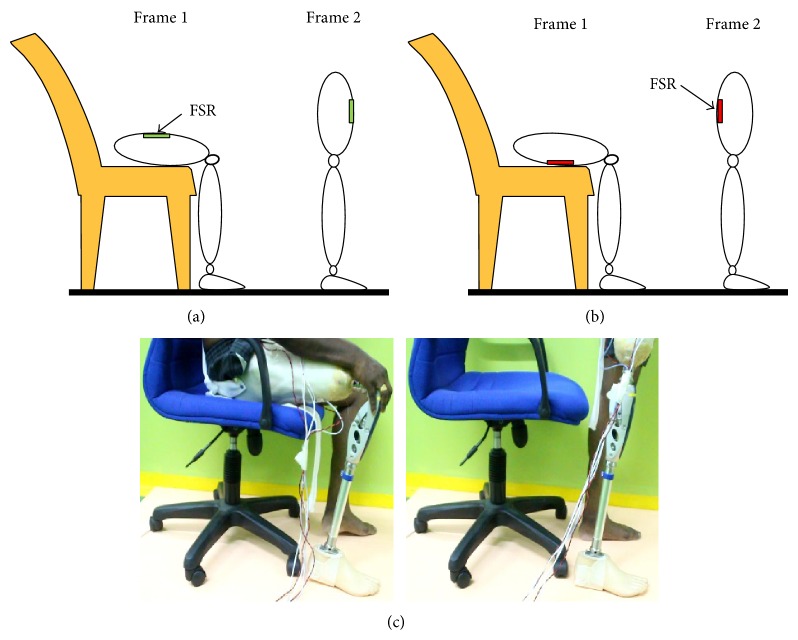
(a) Anterior FSR placement during sit to stand; (b) posterior FSR placement during sit to stand; and (c) the individual performing sit to stand while wearing the FSR instrumented socket.

A 29-year-old male, 75 kg, of height 182 cm transfemoral amputee who had been using an above knee prosthesis for the past 10 years, was recruited for this study. An informed written consent was attained from the subject as approved by the ethics committee of University Malaya Medical Centre. Two separate experiments with the same procedure were performed for each sensor, that is, FSR and piezoelectric sensors. In the first experiment, FSR sensors were attached at the regions of the quadrilateral double socket based on the subject's anatomical muscle position. The quadrilateral double socket was selected as it was the type of socket he had been using thus ensuring no compensatory gait deviations of using a new socket type. The sensors' wires were carefully secured and lengthened to ensure that the participant's movement was not affected. The amputee was fitted with the instrumented socket and knee prosthesis and was requested to perform five repetitions each of complete gait cycle, stair ascent, and sit to stand movements. The subject performed the stance phase of the gait cycle, that is, heel strike, flat foot, and toe off, as shown in Figure 4 for 5 repetitions. The subject was then requested to go for stair ascent by positioning his leg in a flexed position upon an elevated step of 250 mm height (Figure 5), afterwards applying a downward force upon instruction. Finally, the amputee performed sit to stand action. The subject initially sat on a chair and stood up upon instruction (Figure 6).

### 2.3. Signal Processing and Movement Characterization

The signals generated from the user's activities were displayed and processed using Simulink (Real Time Windows Target Toolbox). The envelopes of the gait cycle curves were time aligned with the motion capture to define “heel strike,” “flat foot,” and “toe off” and processed to attain the amplitude patterns. The knee angle at each event was used as a reference to relate it with the captured signals as well as to show the ability of the sensors in characterizing the knee movement. Knee angle was captured by using Kinovea software and measured at each movement 30 Hz sampling rate in order to provide reference platform about the change during different phases. The curve profiles of the various movements were then characterized according to the standard deviation at specific points of each movement.

## 3. Results and Discussion

Variation of the captured signals versus time for FSR and piezoelectric sensors is presented in the following subsections. Knee angle was used as a reference for each case to relate the variation of the sensors output signals with the behavior of each knee movement phase.

### 3.1. Measurements of FSR and Piezoelectric Sensors throughout a Gait Cycle

This study protocol used FSR and piezoelectric sensors separately. A tethered FSR and piezoelectric sensors have been used. Using both sensors tethered together would add to the complexity of the setting which would inherently cause discomfort to the amputee subject thus producing unnatural gait.

**Figure 7:**
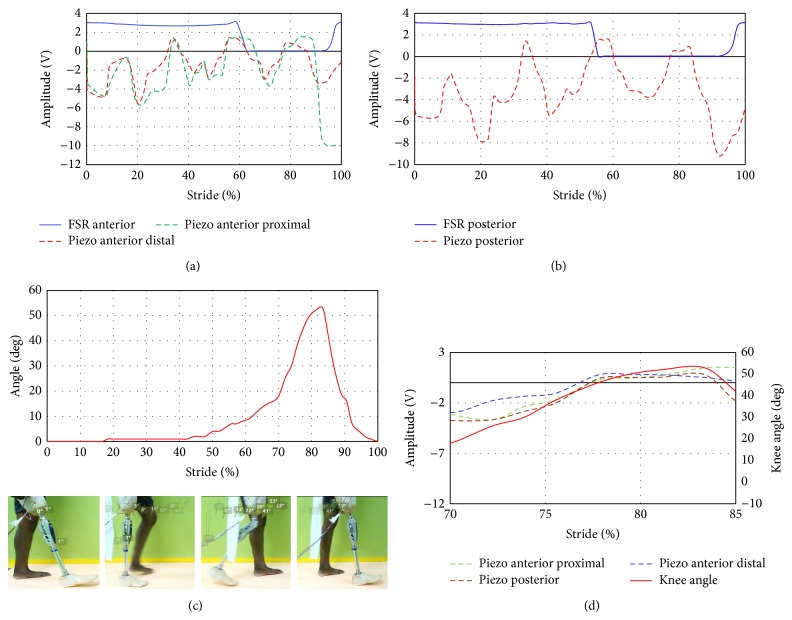
FSR and piezoelectric sensors output during gait cycle: (a) FSR anterior, piezo anterior distal, and piezo anterior proximal sites, (b) FSR and piezoelectric sensors at posterior sites, (c) knee angle during stride, and (d) piezoelectric sensors with the knee angle at a region from 70% to 85%.

The resulting FSR and piezoelectric sensors signals when performing different movements were compared.  Figure 7 shows the FSR anterior and posterior outputs versus the knee angle throughout the gait cycle. The amplitude of both anterior and posterior sites started at heel strike. The pressure generated at anterior/posterior regions were the same as it produced output voltage of 3-3.1 V. However the knee angle at that phase is fully extended to begin the gait cycle. At about 33% of gait the FSR anterior output reached an amplitude of about 2.7 V. However voltage at the posterior sites remained higher than 3 V. At foot flat of 44% from the gait, the anterior voltage starts to increase and the posterior voltage has the same value of about 3 V. In addition, the knee angle started to flex before the time of foot flat preparing for the toe off stage. At the swing phase region which shows the maximum knee flexion of about 53 degrees the FSR output of both anterior/posterior sites displayed minimum of about zero reading which indicates that there is no loading at both sensors at this stage. The gait cycle ended by reaching the full extension of the knee angle and increased the amplitude of anterior/posterior sensors up to 3 V. In essence, FSR could provide information about the gait change from the stance phase to the swing phase as can been seen from anterior/posterior graphs with the knee angle.

Results corresponding to the piezoelectric tests are conducted to be compared with FSRs' trials. Figures 7(a) and 7(b) showed that both anterior distal and anterior proximal sensors have the same trend line at 0–0.4 s of about 0–40% stride. The peaks of piezoelectric sensors demonstrated how the piezoelectric contracted once the pressure was exerted (positive peaks) and released when the piezoelectric retracts (negative peaks). As can be noticed from the knee angle lines during the swing phase at 1.6 s about 70% stride, the trend of both anterior proximal and posterior sensors matches the knee angle; moreover the posterior sensor exhibits similar behavior with the knee angle until the time reached 2 s. The behavior of the posterior piezoelectric sensor mostly had the same trend compared to the knee angle particularly at the swing phase. The toe off stage occurred at about 74% of the gait cycle, while the output voltage from the piezoelectric sensors intersected with neutral at zero voltage. This is because the generated pressure at this phase decreases due to unloading of the subject's leg from the ground. At the end of the gait cycle the output voltage became 10 V and 9 V at anterior proximal and posterior sites, respectively.  Figure 7(d) illustrates the knee angle and piezoelectric sensors signals in the same graph. As illustrated in Figure 7(d), the trend of the piezoelectric sensor at swing phase (75%– 85%) matches the knee angle behavior and the peaks cross the zero to the positive region.  Figure 7(d) shows a closer look at the swing phase region from 70% to 85% to show agreement between the knee angle and the piezoelectric sensors.

### 3.2. Measurements of FSR and Piezoelectric Sensors during Sit to Stand

**Figure 8:**
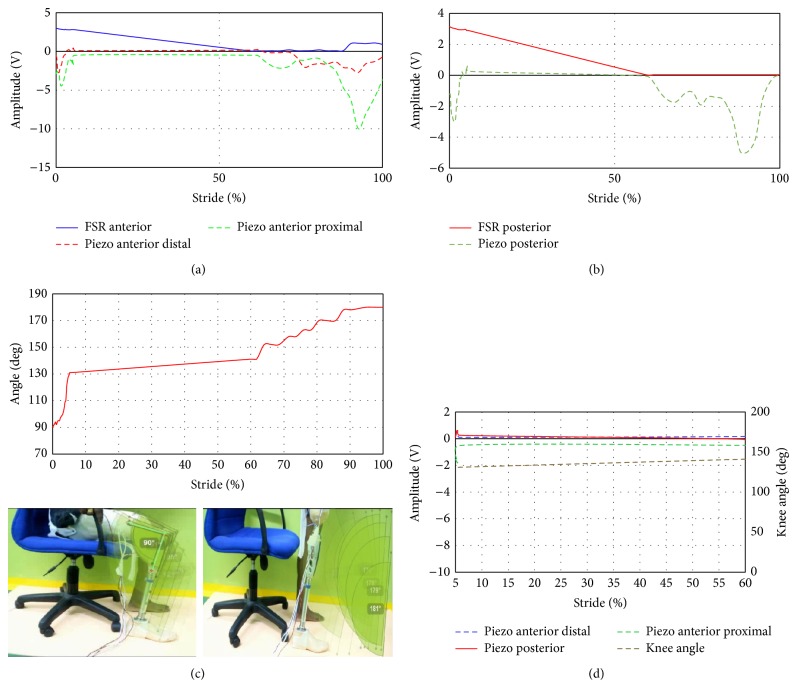
FSR and piezoelectric sensors output during sit to stand movement: (a) FSR anterior, piezo anterior distal, and piezo anterior proximal sites, (b) FSR and piezoelectric sensors at posterior sites, (c) knee angle during stride, and (d) piezoelectric sensors with the knee angle at a region from 5% to 60%.

Similarly, FSR and piezoelectric sensors were used to measure the dynamic variation inside the socket during sit to stand movement.  Figure 8 illustrated the FSR output versus the complete stride during sit to stand. The knee angle shown as a reference (Figure 8(c)) at the start of the sitting position was about 90 degrees opposite to amplitude of 3 to 3.1 V from both anterior and posterior FSR. The knee angle increased to 130 degrees at 5% of the movement. However the output of FSRs decreased below the 3 V, due to the pressure decrease at both anterior/posterior sites compared to the sitting position. The knee angle increased gradually to 180 degrees and consequently the anterior/posterior FSR sensors decreased linearly to the minimum value of about 0 V. Linear decrease of the FSR can be interpreted due to the sudden change of the movement by the subject which started from the sitting position to about 60% of the full stride before the full standing. This is one of the limitations of the FSR during that movement that should be considered in the future applications.

Sit to stand movement was tested and piezoelectric measurements versus stride were presented in Figure 8. The output signals from both anterior distal and posterior meets up from 50% to 60% have a zero voltage value, while at 60% to 100% of the stride, the piezoelectric sensor started to be decompressed as the voltage indicates negative value at that region. At anterior and posterior sites, two peaks of about 10 V and 5 V, respectively, can be noticed before the full standing position of the subject. As can be seen in Figure 8(d), a specific region from 5% to 60% was studied to show the relation between the knee angle and the piezoelectric signals. It is clear that the four signals of sensors and knee angle are straight line of about zero voltage for piezoelectric sensors and linear line of angle of a value of 140 degrees.

### 3.3. Measurements of FSR and Piezoelectric Sensors during Stair Ascent

**Figure 9:**
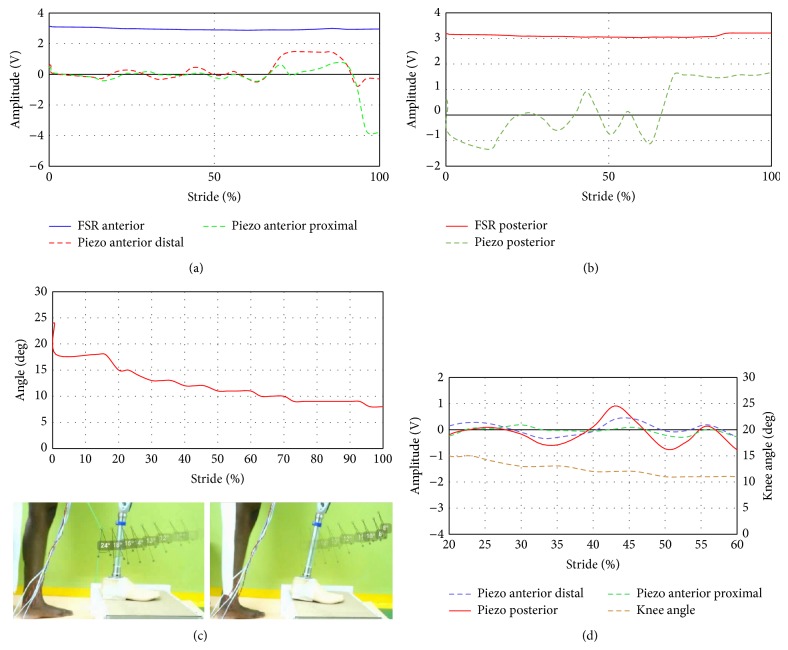
FSR and piezoelectric sensors output during stair ascent: (a) FSR anterior, piezo anterior distal, and piezo anterior proximal sites, (b) FSR and piezoelectric sensors at posterior sites, (c) knee angle during stride, and (d) piezoelectric sensors with the knee angle at a region from 20% to 60%.

Stair ascending was carried out as shown in Figure 9. The foot was placed on the step as shown in Figure 9 before the measurement of knee angle and sensors is started. As illustrated in the graph, the output voltage of both anterior and posterior sensors remains almost constant during the whole event because of the pressure generated from the ground, which is directly reflected as voltage of about 3–3.2 V. The knee angle varied from 23° to 9° at the end of the stair ascent phase. Stair ascent movement was conducted with the user wearing the socket embedded with the piezoelectric sensors. The knee angle decreases gradually from about 23° to 8°; however the variation of the output signals from piezoelectric sensor at both anterior distal and posterior proximal sensors changed minimally during the 0% to 60% stride. Piezoelectric sensor at the posterior site decompressed at the early stage of the stride at 10%. High compression value was noticed at anterior distal site which has a value of about 1.5 V (Figure 9). In overall, Figure 9(d) shows the three piezoelectric signals with the knee angle in the same graph. It can be noticed that the fluctuations of the piezoelectric sensors agreed at a region starting from 20% to 60%. This region can provide information when compared with the variation of the knee angle which starts from 15° to almost 10°.

Analysis was conducted to identify the events during the gait cycle based on the events as described by Nordin and Frankel [22]. The swing phase is divided into initial swing (60–73% of gait cycle), midswing (73–87% of gait cycle), and terminal swing (87–100% of gait cycle). FSR output signals showed some delay during the transition from stand to swing as a result of the FSR characteristics reported that it has 1-2 ms mechanical rise time delay [17]. Therefore, at the walking phase the results of FSR are considered with the mentioned delay and piezoelectric sensors can function better than FSR. Results of the piezoelectric sensors (Figure 8(d)) can be combined to describe midswing and terminal swing events. Figure 9(d) illustrates good agreement between knee angle and the piezoelectric sensors within a range of voltage from −4 V to −2 V and the knee angle proportionally changed from 20° to 55°. Sit to stand phase is important to the transfemoral amputees and the movement events can be identified from the signal pattern. The prestanding phase at 50 to 60% of the movement can be recognized from both FSR and piezoelectric signals (Figure 9).

**Figure 10:**
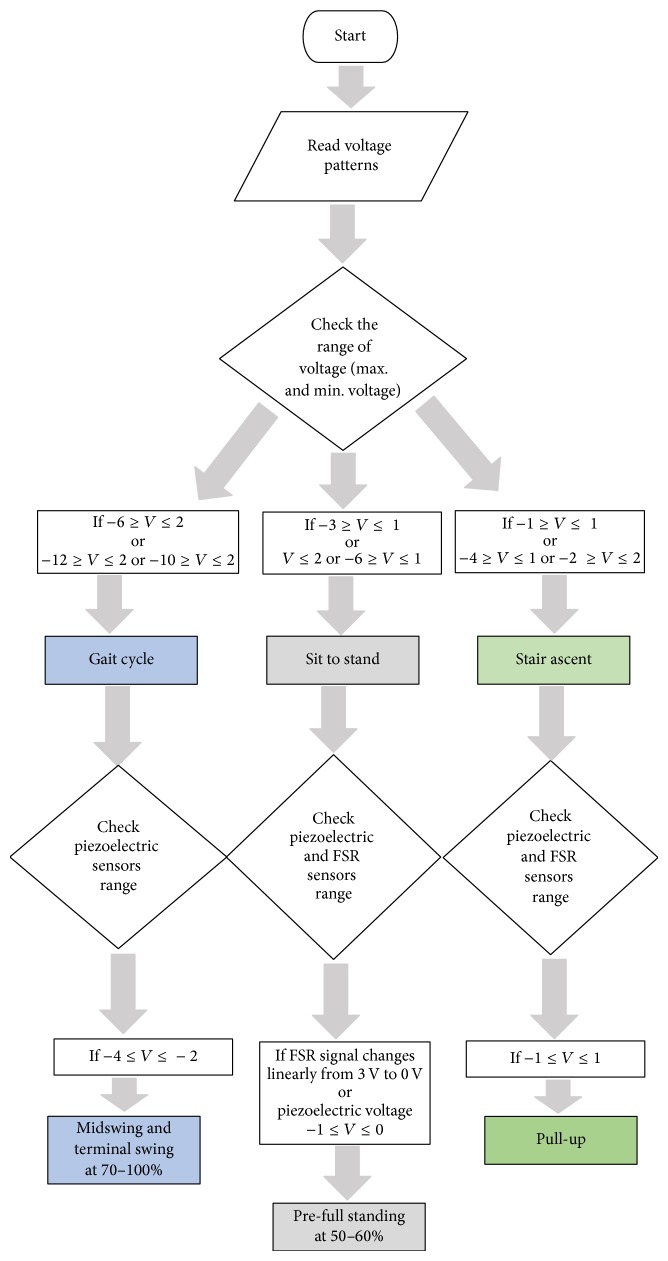
Flow chart presents the identification process of the knee state by consideration the range of voltage of piezoelectric sensor.

Stair ascent movement was divided into five submovements [23]. The pull-up submovement could be determined by considering the piezoelectric signals while its voltage was between −1 and 1 V (Figure 9). Flowchart shown in Figure 10 concludes how the results conducted from the current study are used to build an algorithm to identify specific events during different knee movement. Walking gait, sit to stand, and stair ascent can be identified according to the flow chart. Specifically, midswing and terminal swing can be recognized. At sit to stand movement, pre-full standing event can be seen at 50–60% of the stride. Finally, pull-up event can be identified at the stair ascent movement. The variation of both FSR and piezoelectric sensors readings at specific points during each movement was reflected as the standard deviations in Appendix Tables 2 and 3. As can be noticed the wide range of measurements of piezoelectric sensor will help to identify the knee movement.

## 4. Study Limitation

This study was performed to establish the proof of concept with a single amputee subject particularly to look at the different sensor responses. The session was conducted with five trials per movement represented by the standard deviation values at the Appendix section. To ensure natural walking, quadrilateral socket was used in this study as it is the type of socket that is used by the subject in his daily activities. It was also assumed that the middle of the muscle belly is the area of greatest pressure within the socket, and in this case study it was verified by the greatest pressure felt during the subject's maximum voluntary contraction through manual palpitation of the muscles. In other cases, it could depend largely on the socket fit; thus this factor should be taken into consideration in further studies. Additionally, the current study indicated that the piezoelectric sensors could be useful in recognizing the knee movement better than the FSR because of the variations shown during each phase. More experiments should be conducted with different socket types in order to make better comparison between both sensors used in the current study. Moreover, statistical significance can be obtained by considering more than one subject to make the results more convincing.

## 5. Conclusion

This study presented the possibility of identifying the submovement of a transfemoral amputee using FSR and piezoelectric sensors integrated into the socket. A pair of FSR and three piezoelectric sensors were embedded separately at anterior and posterior sites inside of the socket to be directly in contact with the residual limb of a transfemoral amputee. Complete gait cycles as well as stair ascent and sit to stand motions were performed by the transfemoral amputee to determine the predictability of the knee movement detection as well as user intention by using FSR and piezoelectric sensors. This would be useful in further studies related to the prosthetic knee development. The piezoelectric sensors indicated wide range of measurements at all conducted movements. In particular, piezoelectric sensors can identify submovements at gait and stair ascent movements within a specific range of output voltages. In addition, signals from piezoelectric sensors show acceptable agreement while tracking the knee angle at gait cycle and sit to stand. However, more work should be considered for using piezoelectric sensors at stair ascent/descent and slope climbing. In case of FSR, it could be useful in detecting the change of gait from stance phase to swing phase. FSR showed that it could be used in identifying the pre-full standing phase at sit to stand movement. Therefore, one of the recommendations from this study is that FSR may be more useful to be used as a trigger between the knee movements (walking, sit to stand, and stair ascent) due to its measurement limitations and would complement the piezoelectric signal for major movement detection.

Following this efficacy study, it can be concluded that the user's intended movement could be detected prior to its angular mechanical change using an instrumented socket. Further trials are to be conducted with greater sample size to determine the consistency and accuracy of response in different subjects with different residual limb lengths, socket types, and muscle condition. This study also demonstrated that piezoelectric sensors could be safely and effectively be embedded onto the socket wall to provide reliable response signal that may be helpful in recognizing the user intention and maintain the amputee's comfort and normal stride while wearing his prosthesis. However, more subjects and simulation of different sensing methods are recommended to address more variations in sensor responses. The proposed approach presented in this study could serve as a complementary input to optimize the interaction of the user with the existing or new microcontrolled prosthetic devices.

## Figures and Tables

**Table 1:** Sensory mechanisms used in prosthetic knee systems.

Author (year), system	Sensor type	Mechanism and function
Kapti and Yucenur (2006) [5], artificial knee joint	Rotary knee angle's potentiometer	Detects different angles of the knee joint from 119.5° to 180° as the sensor located at the joint centre.

Sup et al. (2009) [6], Vanderbilt prosthetic leg	Load cell	Detects force and torque loading at the knee and ankle.
	Rotary potentiometer	Detects the knee joint angles.

Martinez et al. (2009), agonist-antagonist prosthetic knee	Rotary encoder Digital encoder, to measure Ankle Angle Digital encoders, to measure motor displacements Hall sensor, to measure springs' Compression Force sensitive resistor, to Heel/Toe Contact	Detects the joint angles by controlling the motor displacement via the rotary encoder, attached to the motor shaft.

Sup et al. (2009) [6], Vanderbilt prosthetic leg	Custom load cell	Custom load cell was made to detect force and torque loading at the knee and ankle.
	Potentiometer	Detects the knee joint angles.

Geng et al. (2010) [4], four-bar linkage prosthetic knee	Knee angle sensor used to detect angle at different phases.	Prosthetic knee with four-bar linkages mechanism

**Table 2:** Standard deviation values for FSR.

	Gait cycle	Standard deviation	Sit to stand	Standard deviation	Stair ascent	Standard deviation
	%	±	%	±	%	±
Anterior	0	0.016	0	0.032	0	0.058
	0.17	0.026	0.23	0.041	0.3	0.074
	44.44	0.047	0.5	0.046	0.6	0.099
	46.11	0.064	59.4	0.029	30	0.165
	62.78	0.000	61.7	0.027	33	0.173
	64.44	0.000	64	0.025	36	0.180
	66.67	0.000	66.3	0.029	40	0.189
	70	0.000	97.7	0.028	96	0.159
	72	0.000	100	0.024	100	0.151
	97.78	0.047				
	100	0.018				

Posterior	0	0.025	0	0.020	0	0.005
	0.17	0.027	0.23	0.010	0.3	0.015
	44.44	0.0 ± 04	0.5	0.013	0.6	0.029
	46.11	0.124	59.4	0.075	30	0.012
	62.78	0.000	61.7	0.078	33	0.014
	64.44	0.000	64	0.081	36	0.015
	66.67	0.000	66.3	0.082	40	0.000
	70	0.000	97.7	0.030	96	0.017
	72	0.000	100	0.027	100	0.019
	97.78	0.004				
	100	0.019				

**Table 3:** Standard deviation values for piezoelectric.

	Gait cycle	Standard deviation	Sit to stand	Standard deviation	Stair ascent	Standard deviation
	%	±	%	±	%	±
Anterior distal	0	0.448	0	0.783	0	1.276
	0.16	1.046	0.22	1.123	0.3	1.102
	42.22	3.057	0.45	3.707	0.6	1.202
	44.44	3.278	5.71	0.252	40	0.138
	46.11	4.086	59.42	0.422	43	0.006
	72.22	2.873	61.71	0.892	46	0.207
	73.88	0.192	64	1.203	50	0.203
	97.77	0.963	66.28	1.694	53	0.224
	100	0.811	97.71	2.149	96	1.308
			100	2.346	100	1.336

Anterior proximal	0	0.849	0	4.617	0	0.654
	0.16	0.712	0.22	3.616	0.3	0.703
	42.22	4.909	0.45	2.428	0.6	0.705
	44.44	3.975	5.71	2.264	40	0.603
	46.11	6.115	59.42	3.961	43	0.044
	72.22	6.031	61.71	4.918	46	0.117
	73.88	5.598	64	5.075	50	0.479
	97.77	1.096	66.28	3.194	53	0.476
	100	1.173	97.71	1.853	96	0.497
			100	1.833	100	0.598

Posterior	0	2.375	0	2.026	0	1.439
	0.16	5.215	0.22	1.650	0.3	1.129
	42.22	5.284	0.45	3.168	0.6	1.442
	44.44	6.136	5.71	2.639	40	0.141
	46.11	5.715	59.42	3.643	43	0.189
	72.22	2.853	61.71	4.032	46	1.253
	73.88	1.628	64	3.375	50	1.270
	97.77	6.081	66.28	3.119	53	1.308
	100	6.048	97.71	4.917	96	1.654
			100	4.930	100	1.675

## Appendix

See Tables 2 and 3.
